# Family members' beliefs and attitudes towards visiting policy in the intensive care units of Ghana

**DOI:** 10.1002/nop2.234

**Published:** 2019-01-07

**Authors:** Yakubu H. Yakubu, Maryam Esmaeili, Elham Navab

**Affiliations:** ^1^ Intensive Care Nursing, School of Nursing and Midwifery Tehran University of Medical Sciences Tehran Iran; ^2^ Nursing and Midwifery Care Research Center Tehran University of Medical Sciences Tehran Iran; ^3^ Department of Critical Care Nursing, School of Nursing and Midwifery Tehran University of Medical Sciences Tehran Iran

**Keywords:** attitudes, beliefs, family, Ghana, intensive care unit, visiting policy

## Abstract

**Aim:**

This study aimed to investigate family members' beliefs and attitudes towards the visiting policies of intensive care units (ICUs).

**Design:**

It employed a descriptive cross‐sectional quantitative design.

**Method:**

This study recruited four public hospitals in Ghana with a sample of 200 family members. The study was conducted using a self‐administered questionnaire. The data were collected and analysed with SPSS version 16.

**Results:**

This study revealed that while family members believed in the beneficial effect of adhering to open visiting policies in ICUs, their attitudes were sceptical and restrictive. Most family members preferred the acceptable number of visitors within 24 hr to be two, and according to them, only one person should be allowed to enter at a time. There was a meaningful relationship between the families' beliefs and religion (*p* = 0.02), educational level (*p* = 0.03) and family status (*p* = 0.02). Furthermore, a meaningful relationship was also observed between the families' attitudes and status (*p* = 0.04) and their level of education (*p* = 0.05). The studied family members showed concern in this regard and did not want the community style of visiting to be implemented, which could hinder patients' recovery.

**Why is this research or review needed?**

- A crucial part of a person's health is the social factor concerning his/her family members and significant others who can help coordinate patient care.
- A comprehensive understanding of the evolution of today's visitation policies and practices can aid in elucidating the context of change in addition to the everyday challenges of developing, recommending and implementing new guidelines on family visitation, presence and participation.
- Strict visiting restrictions reflected concern and a lack of information about the effects of visiting on patients and their families.
- ICU visitation is emerging as an important phenomenon in Ghana.
- Ghana is an underrepresented setting in scientific research concerning critical care.

**What are the key findings?**

- A well‐developed policy for visitation schedules on admission should be in place so that each family has a plan that suits its unique dynamics.
- In our study, family members had sceptical attitudes towards open visiting in ICUs. Hence, they need support to overcome their perceived fears concerning the culture of visiting the sick and dying, which exist in their communities.
- The family members recognized the benefits of open visitations. However, they held sceptical and restrictive attitudes towards such a policy concerning the numbers of family members allowed to visit at a time in conjunction with the critical and emotional state of the patient. This was primarily caused by environmental factors and cultural influences and beliefs.

**How should the findings be used to influence policy/practice/research/education?**

- Understanding families' beliefs and attitudes about visiting policy at ICUs, through the findings of this study, will allow stakeholders to suggest changes in the existing policies to safeguard patient care to attain greater satisfaction among family members in our critical care wards.
- Cultural dynamics, which are critical according to the families, should be taken into consideration when recommending a visiting policy.

## 1 BACKGROUND

Family plays an essential role in the care and recovery of patients admitted to intensive care units (ICUs). The ICU is a stressful environment for both patients and their families (da Silva Ramos, Renata, Azevedo, & Schettino, 2013). The admission of patients to the ICU is usually instantaneous. Thus, families are usually unprepared for such a circumstance and consequently experience anxiety (Hojat, Tabandeh, & Fatemeh, 2016). Recent studies have identified several stressors present in an ICU environment such as fear of unknown consequences, routine disruption, unfamiliarity with the environment and emotional upheavals (Auriemma et al., 2015; Fumis, Ranzani, Faria, & Schettino, 2015). These factors often serve as a source of psychological distress for the family (Maité et al., 2008). Furthermore, nurses are also not spared from working in this stressful environment while remaining constantly aware of the fact that another patient can come through the door at any minute (Melissa, 2012). The stresses experienced by nurses and family members can result in a lack of communication, poor mannerisms and dissatisfaction among family members, especially with respect to visiting their loved ones (da Silva Ramos, Lins Fumis, Luciano, & Schettino, 2014).

Globally, until the recent past, the ICU has been a restricted environment for visitors and families of patients. Family members consistently preferred being closer to the patient, whereas these units have had traditionally restricted areas (Baning, 2009). Owing to scientific and technological progress, ICUs have undergone numerous changes including the use of highly advanced equipment, which are both invasive and non‐invasive, in monitoring patients (Cappellini, Bambi, Lucchini, & Milanesio, 2014). Findings from multiple studies have attested to the beneficial effects of open visitation, which include decreased patient anxiety, increased level of family satisfaction, improved communication between healthcare providers and families and increased patient rest. Moreover, the importance of proximity to the patient was rated to be high (69%) by family members in the study conducted by Gundo, Bodole, Lengu, and Maluwa (2014) where the perceptions concerning family needs in critical care units were studied. Furthermore, most families believe that the patient felt safer and more supported if they were present (McAdam & Kathleen, 2013).

Flexible visitation policies can help in creating a healing environment where optimal patient outcomes are achieved. The American Association of Critical‐Care Nurses (AACN) Practice Alert (2011) shows that flexible visitation policies improve patients' general safety and communication in addition to the level of satisfaction of family and staff members. Moreover, the other advantages in this regard include calming effects, support and comfort drawn from the presence of family members. Research also suggests that a flexible visiting policy increases the satisfaction of patients and their families, reduces their anxiety and helps them integrate more effectively into the complicated and hostile ICU environment (Athanasiou, Papathanassoglou, Patiraki, McCarthy, & Giannakopoulou, 2014; Hart, Hardin, Townsend, Ramsey, & Mahrle‐Henson, 2013).

On the other hand, several other studies have posited that an open visiting policy has numerous positive outcomes for both the patient and their family members. In addition, it has been shown that the presence of visitors in ICUs positively affects patients' outcomes such as the sense of well‐being and the healing process (Fumis et al., 2015; Obringer, Hilgenberg, & Booker, 2012). A randomized study conducted in Italy by Fumagalli et al. (2006) associated open visitation policies with a decrease in patient anxiety, improvements in their hormonal profiles and a decrease in cardiovascular complications. Furthermore, ICU nurses' attitudes towards such policies vary widely and these policies are not always implemented by the nurses who control the access to patients (Athanasiou et al., 2014). According to a study conducted in Turkey by Sevim et al.(2013) with regard to the views of nurses and families in terms of ICU visitation policies, the nurses working at critical care units believed that only first‐degree relatives should visit the ICU and this opinion was reported at a rate of 99%.

A crucial part of an individual's health is the social support extended by his/her family members and significant others. In the African tradition, a family refers to the nuclear as well as the extended unit. African communities are characterized by the prevalence of collectivism as opposed to individuality. Moreover, cohesion, compassion and mutual support for neighbours are held in high esteem in such communities. Hence, a problem faced by a member becomes a collective problem of the community, which includes one's health and sickness (Constantine, 2014).

Traditionally, families in these communities live together in the same extended residential unit. In this arrangement, grandparents, parents and children live in the same compound or nearby and have an equal share in good and bad times. Thus, a person is always surrounded by close ones during a period of difficulty (Yaw & Baffour, 2005). Such family connections serve as a means of social, economic and psychological security in troubled times. Thus, the family is the bedrock of Ghanaian society. It transmits cultural heritage and serves as the first line of social security. In Ghana, there is no guideline or formal recommendation about the visiting policies followed in ICUs. Every institution determines its own individual visitation strategy based on traditional practice, belief or self‐intuition without any empirical evidence.

Based on anecdotal evidence and the first author's experiences in Ghana in ICUs, where nurses are clearly in charge, visitation is commonly viewed as a privilege, not a right; it depends on several factors such as the patient's condition and the particular nurse's beliefs and attitudes. Certain families clearly benefit from selective visitation, whereas others report experiencing stress owing to rigid rules. In case of selective visitation (a form of restricted visiting), a patient's immediate caregiver is required to document a limited number of people who would be unconditionally allowed to visit the patient at a stipulated time (Bettina, White, Graham, & Alexandrov, 2014). A study conducted in Iran by Tayebi, Dehghan Nayeri, and Borimnejad (2017), concerning the dominant strategies adopted about visitation in selected ICUs, concluded that the most suitable strategies implemented to bring about positive impacts of visiting on the process of physical and mental recovery of ICU patients were seemingly related to personalized cultural traits of individuals and individualized visiting. Hence, much of the research conducted since the 1980s has concentrated on nurse's perspectives pertaining to their beliefs and attitudes as well as on the needs of the families of critically ill patients. No literature related to Ghana or even Africa as a whole was found on familial beliefs and attitudes or in relation to their satisfaction with visiting policies and hours. Since, in Ghana, family members are integral to the care of a patient, it is imperative to evaluate the beliefs and attitudes of family members with regard to the visitation policies of ICUs.

## 2 METHODS

### 2.1 Aim

To investigate family members' beliefs and attitudes towards visiting policies in ICUs.

### 2.2 Study design

A descriptive cross‐sectional design was employed in this study, the location of which is Ghana.

### 2.3 Study setting

This study was conducted in four ICUs, two from university teaching hospitals (Komfo Anokye Teaching Hospital in the south and Tamale Teaching hospital in the north) and two from public regional hospitals (Bolgatanga Regional Hospital in the north and Tema General Hospital in the south). The Komfo Anokye Teaching Hospital (KATH), which is situated in Kumasi in the Ashanti Region, is the second‐largest hospital in Ghana with a capacity of approximately 1,000 beds. It has a paediatric ICU and a general ICU, which admits adults and has only eight beds.

On the other hand, the Tamale Teaching Hospital is affiliated to the University for Development Studies' School of Medicine and Allied Health Sciences. It is located at the regional capital, Tamale, in the northern region of Ghana. It serves as a referral hospital for the following three northern regions of Ghana: Tamale, Upper West and Upper East. Furthermore, it also serves a few neighbouring countries such as Burkina Faso, Ivory Coast and Togo. It has a bed capacity of about 800 and a general ICU with a bed capacity of 16.

The Bolgatanga Regional Hospital is situated in the Upper East regional capital and has a General ICU with a capacity of only two beds. The Tema General Hospital is located at the Tema Municipality, which is close to the Tema ports and harbour. It serves all the Tema communities present in the Greater Accra region of Ghana and has a General ICU with a capacity of four beds.

### 2.4 Study participants

In this research, the study population included the family members of patients who had been admitted to ICUs. The sampling procedure that was adopted was simple convenience sampling with a confidence interval of 95% and a power test of 90%. In the frame of sampling, first‐ and second‐degree relatives as well as their confidants aged above 18, who were of sound mental health and were willing to participate in the study, were recruited. Most families in Ghana live under an extended family system, wherein every member resides in the same large house. Moreover, there is no significant difference in terms of family members' beliefs and attitudes. The sample size of this study was 200. The inclusion criteria included family members who had visited their relatives who were admitted to the ICU; they needed to be at least 18 years old and had to be willing to participate in this study. Literacy was not considered in the selection of participants. The exclusion criteria of this study comprised those who visited the patients but never identified themselves as their family. Ghana largely constitutes a typical African society, with a typical communal life. The sense of collectivism means that neighbours who have lived in the same community for a while are often regarded as families and confidants. These connections serve as a means of social and psychological support to community members in times of crisis. To this end, everyone in the vicinity wants to share in the pain and empathize with their neighbours in the event of sickness or ill health.

### 2.5 Data collection

Data were collected between June 2017‒December 2017. The first author was present on‐site to supervise the data collection process. Demographic information concerning the patients' families included seven questions pertaining to gender, age, religion, ethnicity, relationship with patient, employment status and level of education. Moreover, data were collected by means of a standard instrument called Beliefs and Attitudes Visitation Questionnaire (BAVIQ), which has been developed by Berti, Ferdinande, and Moons (2007); permission to use the instrument was granted by Dr. Philip Moons (Academic Centre for Nursing and Midwifery, University of Leuven). The BAVIQ was developed to assess nurses' beliefs and attitudes about the visiting policies adopted in ICUs. Moreover, this permission was sought and granted to eliminate the questions that were nursing focused and those that could not be ascertained by the family members. As a result, five questions were eliminated from the said questionnaire because they were based on the beliefs and attitudes related to the haemodynamics of patients, open visiting policy interference with the relationship between nurses, psychological stress experienced by nurses, nursing processes and visiting policies that make nurses feel in control. It must be noted that these changes did not alter its reliability or validity. Additionally, minor revisions were made to improve the ease of understanding and to reflect the content to ensure suitability for the families' use.

In this study, content and face validity were established. The questionnaire was submitted to five faculty members of the intensive care nursing department of the Tehran University of Medical Sciences and four master's degree ICU nursing candidates for review and to ensure that the tool was suitable to be used for the family members participating in this study.

A pilot study was conducted with a convenience sample of 20 family members whose relatives were admitted to the ICU. This was done to evaluate the metric characteristics of the modified questionnaire (internal consistency, reliability and validity) in addition to the stability of the measurement used by the family members. The English version of the questionnaire was used, and the illiterate family members who could not read were assisted. Each item or line was explained to them to ensure that the content was well understood before they made a choice.

The structured questionnaires comprised 14 items assessing the participants' beliefs and 14 items assessing their attitudes. The questions elicited responses according to a five‐point Likert scale format, which had answers ranging from “strongly agree” to “strongly disagree.” It was a self‐reported questionnaire, and where necessary, the questions were clarified to those who had difficulties in understanding.

The said questionnaire contained both positively and negatively formulated questions. To calculate an overall score of the families' beliefs, a few of the responses were reverse‐coded and the questions were negatively formulated. Subsequently, the average score of all the belief items was computed. A score of zero corresponded with beliefs that are strongly opposed to open visitation, whereas a score of four corresponded with beliefs that are strongly in its favour.

In the scoring system, “agreed” and “strongly agreed” and similarly, “disagreed” and “strongly disagreed” were added for the purpose of determining the belief or the attitude status of the respondents for each item that they responded to. However, the responses stating “neither agree nor disagree” were not computed but were considered to be neutral. A score of at least more than 50% was considered to be of high value and was reported.

### 2.6 Data analysis

Data were analysed with the help of a descriptive and analytical statistical test employing Statistical Package for Social Sciences program (SPSS) version 16. In addition, these observations were computed using a chi‐square test and an independent *t* test. The first and second authors managed the data and analysis.

### 2.7 Ethical considerations

Ethical approval was obtained from the following sources: the Komfo Anokye Teaching Hospital's (CHRPE/AP/573/17) ethical review board; the research and planning units of the Tamale Teaching Hospital (TTH/R&D/SR/17/54), the Bolgatanga Regional Hospital (TTH/R&D/SR/17/54), and the Tema General Hospital (TGH2/1/18); and the Tehran University of Medical Sciences' ethics (IR.TUMS.FNM.REC.1396.2703) and institutional review board. Furthermore, informed consent was obtained from the family members, who were willing to participate in the study.

## 3 RESULTS

A total of 146 out of 200 family members (73%) consented and participated in this study. The study subjects' baseline characteristics included a mean of 28.9 (*SD* 4.72) years old. It reported the minimum and maximum age of the family members to be 23 and 33 years, respectively, with most (61%) of the respondents being females. Most of them were unemployed (74.6%) and had received a tertiary level of education (71.2%; Table 1).

**Table 1:** Families' demographic characteristics (*N* = 146)

Variable	*N* (%)
Gender	
Male	57 (39)
Female	89 (61)
Age	
23–27	77 (52.7)
28–32	51 (34.9)
>33	18 (12.3)
Age (Mean ± *SD*)	28.9 ± 4.72
Religion	
Muslim	67 (45.9)
Christian	79 (54.1)
Employment status	
Employed	16 (11)
Unemployed	109 (74.65)
Other	21 (14.4)
Ethnicity	
Dagomba	59 (40.4)
Asante	39 (26.7)
Ewe	21 (14.4)
Frafra	19 (13)
Other	8 (5.5)
Education	
Secondary	42 (28.8)
Tertiary	104 (71.2)
Family status	
Family head	8 (5.5)
Husband	20 (13.6)
Wife	1 (0.7)
Son	29 (19.9)
Daughter	88 (60.3)

From this study, it was observed that family members who completed the questionnaires showed great support for the benefits of visitations (61%) and the benefits of visiting policies with a mean score of 3.39 (*SD* 0.48). From Table 2, it can be noted that most (65.1%; *N* = 95) of the family members wanted the patient to approve the visitation list. However, they held negative attitudes with regard to adapting the visiting policy as per the culture/ethnicity of the patient (80.8%). Furthermore, the family members reported that the duration of visits should be limited (89%) and that the number of visitors permissible within 24 hr should also be limited (74.4%). To elaborate, they wanted three individuals to be allowed to visit the ICU within 24 hr with a mean of 3.60 and a standard deviation (*SD*) of 2.19. Furthermore, the participants supported the idea that only one person should visit the patient at a time with a mean score of 1.86 and *SD* of 1.03. In addition, they presented negative responses against the strict starting hours included in the visiting policies during hospitalization (67.8%). There was, however, a split view between the family members about the adaptation of a strict visiting policy when the patient suffered from emotional problems and with regard to the notion that a strict visiting policy must not be practiced when the family faces practical problems adhering to the said policy (93.8%). Most (51.3%) of the family members also wanted the patient to control the visitation schedule only when the patient was able to do so. The total mean (*M*) and *SD* score of the family members' attitude towards the visiting policy were computed to be 1.40 and 0.71, respectively.

**Table 2:** Beliefs and attitudes of family members

Beliefs questions	Scores (%)				
	Strongly disagree	Disagree	Neither agree nor disagree	Agree	Strongly agree
I believe that visitation has a beneficial effect on the patient	5.5	19.2	6.2	49.3	19.9
I believe that visitation hinders the patient's rest	2.5	0	17.8	54.0	6.8
I believe that an open visiting policy is important for the recovery of the patient	11	26	19.9	42.5	0.7
I believe that visitation causes psychological stress for the patient	19.9	0	11	69.2	0
I believe that visitors can help the patient interpret information	0	0	5.5	78.8	6.2
I believe that an open visiting policy infringes upon patient's privacy	0	0	21.2	78.8	0
I believe that an open visiting policy offers more comfort to the patient	5.5	40.4	0.7	47.9	5.5
I believe that an open visiting policy decreases family's anxiety	0	0	11	74.7	14.4
I believe that an open visiting policy exhausts family, because they feel forced to be with the patient	13	41.8	6.2	25.3	13.7
I believe that an open visiting policy interferes with direct nursing care of patient	0.7	0	14.4	66.4	18.5
I believe that an open visiting policy makes nurses nervous, because they are afraid to err	27.4	55.5	6.2	5.5	5.5
I believe that an open visiting policy makes nurses to spend more time in providing information to the family	0	21.2	0	65	13
I believe that visitation is a helpful support for the care givers	5.5	0	5.5	88.4	0.7
I believe that an open visiting policy contributes to the improvement of patient‐centred care	0	33.6	5.5	60.3	0.7
Total score * Mean (*SD*)	=3.39 (SD 0.48)				

Family members questions on attitudes	Scores (%)				
	Strongly disagree	Disagree	Neither agree nor disagree	Agree	Strongly agree
I think that everyone is allowed to visit, if it is approved by the patient	5.5	29.5	0	65.1	0
I think that the number of visitors in a time range of 24 h should not be limited	27.4	47.3	0	19.2	6.2
I think that the length of a visit should not be limited	40.4	48.6	5.5	5.5	0
I think that the number of people who are visiting the patient at the same time should not be limited	27.4	48.6	5.5	0	0.7
I think that an open visiting policy should be carried out in our unit	11	55.5	32.9	0	0.7
I think that strict visiting hours must be adapted when the family has practical problems adhering to the policy	18.5	75.3	0	0	6.2
I think that strict visiting hours must be adapted when the patient has emotional needs	18.5	31.5	0	49.3	0.7
I think that when the patient is capable, he/she should have control in when, how long and how many visitors he/she can have	5.5	43.2	0	36.3	15.1
I think that the visiting policy must be adapted to the culture/ethnicity of the patient	17.8	63	0	18.5	0.7
I think that a strict starting hour is important, but the length of a visit can be flexible	5.5	26.7	0	67.8	0
I think that the visiting policy must be flexible during the first 24 h of hospitalization	6.2	48.6	0	45.2	0
I think that the visiting policy must be adapted when the patient is dying	11	75.3	0	13	0.7
Total score * Mean (*SD*)	=1.40 (SD 0.71)				

### 3.1 Relationship between the families' beliefs and attitudes

In this study (Table 3), a *p* value <0.05 indicated a meaningful relationship. So, a meaningful relationship was noted between the families' beliefs and religion (*p* = 0.02), educational level (*p* = 0.03) and family status (*p* = 0.04). Moreover, a significant correlation was also observed between the families' attitudes and family status (*p* = 0.04) and their level of education (*p* = 0.05).

**Table 3:** Relationship between families' characteristics of beliefs and attitudes

Beliefs and attitudes	Demographic characteristics							
	Age	Gender	Employment status	Status in family	Religion	Ethnicity	Education level	Test
Families' beliefs	*χ* ^2^ = 77.7	*χ* ^2^ = 24.56	*χ* ^2^ = 77.38	*χ* ^2^ = 29.98	*χ* ^2^ = 0.09	*χ* ^2^ = 1.19	*χ* ^2^ = 88.9	*χ* ^2^ Test
	*p* = 0.16	*p* = 1.71	*p* = 0.9	*p* = 0.04	*p* = 0.02	*χ* ^2^ = 0.07	*p* = 0.03	
Families' attitudes	*χ* ^2^ = 1.36	*χ* ^2^ = 33.07	*χ* ^2^ = 93.25	*χ* ^2^ = 3.7	*χ* ^2^ = 37.71	*χ* ^2^ = 2.29	*χ* ^2^ = 88.92	
	*p* = 0.12	*p* = 0.8	*p* = 0.26	*p* = 0.04	*p* = 0.09	*p* = 0.08	*p* = 0.05	

## 4 DISCUSSION

There is a general agreement about the importance of adopting an open policy guideline in ICU owing to its benefits for critically ill patients and their family members. The current study is the first to provide baseline data on family members' beliefs and attitudes towards an open visiting policy implemented in ICUs in Ghana. Moreover, it has both regional and global applications for the African subregion. Currently in Ghana, all ICUs have restricted visitation policies, a situation similar to that which exists globally. However, there is growing agreement about more liberal visitation policies with some nations already changing their visiting protocols and guidelines. Furthermore, in Ghana, the time allowed per visit is longer in comparison with that of other countries with more restrictive visiting policy than what we observed in our study. For example, a study in Belgium indicated that about 96.7% of ICUs operated with a restricted visiting policy (Berti et al., 2007).

The findings of this study showed that most of the family members believed in the beneficial effect of an open visiting policy on the patient. However, it indicated that they thought the patients needed rest; otherwise, it results in psychological distress for the patients. They also believed that such a policy could infringe on the patient's privacy. The consensus in this regard was that while a family might feel there are certain benefits of an open visiting policy for the patient, they were also cautious of the opportunity that it could hinder the recovery of the patient. This can be compared to a “flat gate” cultural response, according to which in Ghanaian tradition, families live together in the same extended large compound. As per this arrangement, grandparents, parents and children live in the same compound or in a nearby one, sharing good times and bad times alike. Hence, one is always surrounded by several family members during a period of difficulty (Yaw & Baffour, 2005). This could hinder a patient's rest, especially when so many people turn up to visit. Furthermore, there are spiritual and superstitious connections to this approach as well since some of those who come to visit the patient do not necessarily have the best interests of the patient at heart.

Despite the aforementioned observations, from our findings, it can be concluded that the participants believed that an open visiting policy was very important for the recovery of a patient as it offers more comfort to the patient and decreases the family members' anxiety. They also believed that it could offer visitors an opportunity to interpret information for patients since the latter could trust and feel more comfortable with their family members who understood them better.

Several studies, conducted internationally, support our findings; open visitation increases family members' satisfaction, decreases their anxiety, promotes better communication and contributes to the better understanding of a patient (Fumagalli et al., 2006; Maité et al., 2008; Marco et al., 2006). Furthermore, a consistent finding in this regard was the belief that allowing one's family in the ICU or including them in the healing process enhanced nursing care owing to the valuable information obtained in the process (Berti et al., 2007; Kirchhof, Pugh, Calame, & Reynolds, 1993). Visitors provide information that supports healthcare professionals to better understand the patient's personality and coping style (Gonzalez, Carroll, Elliott, Fitzgerald, & Vallent, 2004).

Our study showed that these Ghanaian family members did not believe that an open visiting policy exhausted the family or that it made nurses frightened of being held accountable for their mistakes. In contrast, they believed that open visitation could be a burden for nurses providing care in terms of essential communication and direct nursing care. Most of the family members also believed that such visitations served as a means of helpful support for caregivers and that an open visiting policy contributes to the improvement of patient‐centred care. This is probably because open visitation allows for more opportunities pertaining to patient and family health education since the family becomes more involved in patient care. Numerous studies conducted in other countries support the beliefs of the family members, stating that restricted visiting policies are preferred by healthcare professionals, especially by nurses, because according to them, opening an ICU to visitors could interfere with their care process and organization of care (Beesley et al., 2016; Biancofiore, Bindi, Barsotti, Menichini, & Baldini, 2010).

In our study, most of the families wanted visitation to be approved by the patient. This is probably because they wanted the visiting policy to be based on the patient's need. This is supported by many studies asserting that patients' visiting policy followed in ICUs must be designed on the basis of the patients' and their families' needs (Khaleghparast, Joolaee, Ghanbari, et al., 2016; Khaleghparast, Joolaee, Maleki, et al., 2016). The findings derived from our study indicated that the family members wanted the duration of visits to be limited; they also wanted the number of people visiting the patient at the same time to be limited in addition to the number of visitors during a 24‐hr period. The consensus in this regard was that while an open visiting policy was preferred, the participants did not want too many visitors to interfere with patient care. This is because Ghanaian culture reinforces social cohesion during critical illness or bereavement amidst relatives who do not normally get along too well. Moreover, this is because families in Ghana live in an extended and larger environment, wherein every member of these large families would want to visit the patient, which could be bothersome to a patient. Haghbin, Tayebi, Abbasian, and Haghbin (2011) suggested that visiting policies should be revised to create a more positive environment and provide greater satisfaction to the patients and their families. Accordingly, they do not recommend the general and universal implementation of open ICU visiting policies but propose a modification in the policies on the basis of the cultural backgrounds of Iran, such that a balance is established between the safety of the patients and the needs of their families.

From the current study, it was noted that the family members did not want visiting policies to be strict when visitors experienced practical problems adhering to the said policies. However, most of them wanted a strict visiting policy to be adapted when the patient was receiving a high degree of critical care and facing severe emotional problems. They, however, wanted a restrictive policy for only the immediate family members during an emotional crisis. This prevents the culture of allowing a large number of visitors from one's extended family, who might pose a threat to the focus on the recovery of the patient. Internationally, healthcare professionals have always adopted a somewhat flexible policy when visitors experience practical problems adhering to the hospital policies and/or when a patient suffers from emotional problems (Baning, 2009; Berti et al., 2007). Furthermore, the participants wanted patients to have control regarding the duration of visits and number of visitors that he/she can have at a time. About 80.7% of the participants did not want the visiting policy to be adjusted to the culture/ethnicity of the patient. This is because doing so would not work effectively, considering the multi‐ethnic, multireligious and multicultural society of Ghana.

Moreover, the family members thought that a strict starting hour is important, but the length of a visit needed to be flexible, especially during the patient's first 24 hr of hospitalization. This is important for the family members as ICU admissions are often unexpected and families are typically unprepared and stressed. A flexible visiting policy for the first 24 hr of hospitalization will lessen families' anxieties and worries for their loved one. Another interesting finding in this regard is that despite the fact that family members desired a flexible visiting policy, they did not think that the current visiting policy needed to be adapted in cases when a patient was dying. Ghanaians protect the secrets of the dead; the cultural imperative is to not allow other visitors to see the way their relative passed away. Surprisingly, the current finding stands in contrast to the international research conducted in this regard, pertaining to whether ICU healthcare staff members are in favour of an adjustment in the visiting policy in end‐of‐life situations and/or when the patient is dying (Berti et al., 2007; da silva Ramos et al., 2014).

## 5 CLINICAL IMPLICATIONS

According to this study's observations as well as the review of the current literature, Ghanaian ICU visitation policies should be made more flexible and open due to the beneficial effects of visitations experienced by patients. A well‐developed policy for visitation schedules on admission with the family members should be set in place so that each family can develop a plan that suits its unique dynamics. In our study, family members held sceptical attitudes towards the adherence of an open visiting policy in ICUs. Hence, it can be concluded that they need support to overcome the perceived fears about certain cultural taboos pertaining to visiting the sick and dying, which exist in their communities. Future qualitative studies considering the ethnographical and cultural differences about visiting policies are recommended.

## 6 LIMITATIONS

Even though there was a higher sample estimate initially, only four hospitals constituted the final sample. The response rate to the questionnaire was 73%, which is acceptable for long questionnaires in general and for distressed family members. However, it is fairly low in comparison with the said rate of 90% suggested in the literature. The length of the questionnaire could account for this response rate. Lastly, the difficulty experienced in obtaining ethical clearance, to include the largest metropolitan hospital of Ghana in this study, could also affect its generalization.

## 7 CONCLUSION

Based on the findings of this study, Ghanaian family members surveyed the preferred open visitation policies in ICUs. They recognized the benefits of open visitations. However, they held sceptical and restrictive attitudes towards such a policy with respect to the number of family members permitted to enter the ICU at a time in conjunction with the critical and emotional state of the patient. This observation was largely influenced by the circumstantial and cultural influences as well as their personal beliefs.

## ETHICAL APPROVAL

The study was approved by the Institutional Ethics Committee in Tehran University of Medical Sciences Ethics committee (IR.TUMS.FNM.REC.1396.2703) and institutional review board of Komfo Anokye Teaching Hospital Ghana (CHRPE/AP/573/17), Bolgatanga regional Hospital (TTH/R&D/SR/17/54), Tema General Hospital (TGH2/1/18).

## CONFLICT OF INTEREST

The authors declare that they have no competing interests.

## AUTHORS CONTRIBUTIONS

YHY, EN and ME: Study design. YHY: Data collection and Manuscript preparation. ME: Study design and statistical analysis. EN: Review and proofreading. All authors contributed to the conception and design, acquisition of data or analysis and interpretation of data. All authors read and approved the final manuscript.
